# Incidentally diagnosed multiple intradural extramedullary spinal hydatidosis in a young adult: A case report and review of the literature

**DOI:** 10.1002/ccr3.7691

**Published:** 2023-07-10

**Authors:** Seyyed Mostafa Ahmadi, Keyvan Tayebi Meibodi, Neda Raeesi, Mohammad Ali Bitaraf, Arad Iranmehr

**Affiliations:** ^1^ Department of Neurosurgery, Imam Khomeini Hospital Complex Tehran University of Medical Sciences Tehran Iran; ^2^ Tehran University of Medical Sciences Tehran Iran; ^3^ Department of Neurosurgery, Sina Hospital Tehran University of Medical Sciences Tehran Iran

**Keywords:** Echinococcus granulosus, hydatid cyst, spinal hydatidosis

## Abstract

**Key Clinical Message:**

Although quite rare, vertebral hydatidosis should always be considered as a differential diagnosis for spinal presentations, particularly in endemic areas for echinococcosis.

**Abstract:**

In this paper, we report a rare case of asymptomatic multiple intradural, extramedullary spinal hydatidosis, incidentally diagnosed in a patient with signs and symptoms of a true protruded disc. Although quite rare, vertebral hydatidosis should always be considered as a differential diagnosis for spinal presentations, particularly in endemic areas for echinococcosis.

## 1 INTRODUCTION

The Greek word “hydatid” means “watery cyst.” Echinococcus granulosus, a helminth of the Cestoda class, is the major cause of hydatid disease, a zoonotic infection.^1^, ^2^, ^3^ The World Health Organization (WHO) has identified 20 neglected tropical diseases (NTDs), and echinococcosis is one of them. It affects over a million individuals worldwide and costs the healthcare system more than $3 billion annually.^4^ Most commonly, the liver and lungs are impacted by Echinococcus granulosus. Only 0.2%–1% of patients have skeletal involvement, and 50% of these instances have spinal hydatidosis.^4^ In this paper, we aim to present a rare case of incidentally diagnosed multiple spinal hydatidoses and give a review of the literature about the disease.

## 2 CASE PRESENTATION

A 26‐year‐old male presented with low back pain that radiated to the lower limbs accompanied by bilateral anterior thigh hypoesthesia. The symptoms developed gradually 1 year ago and did not improve with medical treatments. Three years ago, he had cardiac surgery due to cardiac hydatidosis. He took albendazole for about 1 year and a half afterward. He had no history of contact with dogs and livestock and no complaint of coughs, weight loss, night sweats, or other constitutional symptoms.

**FIGURE 1:**
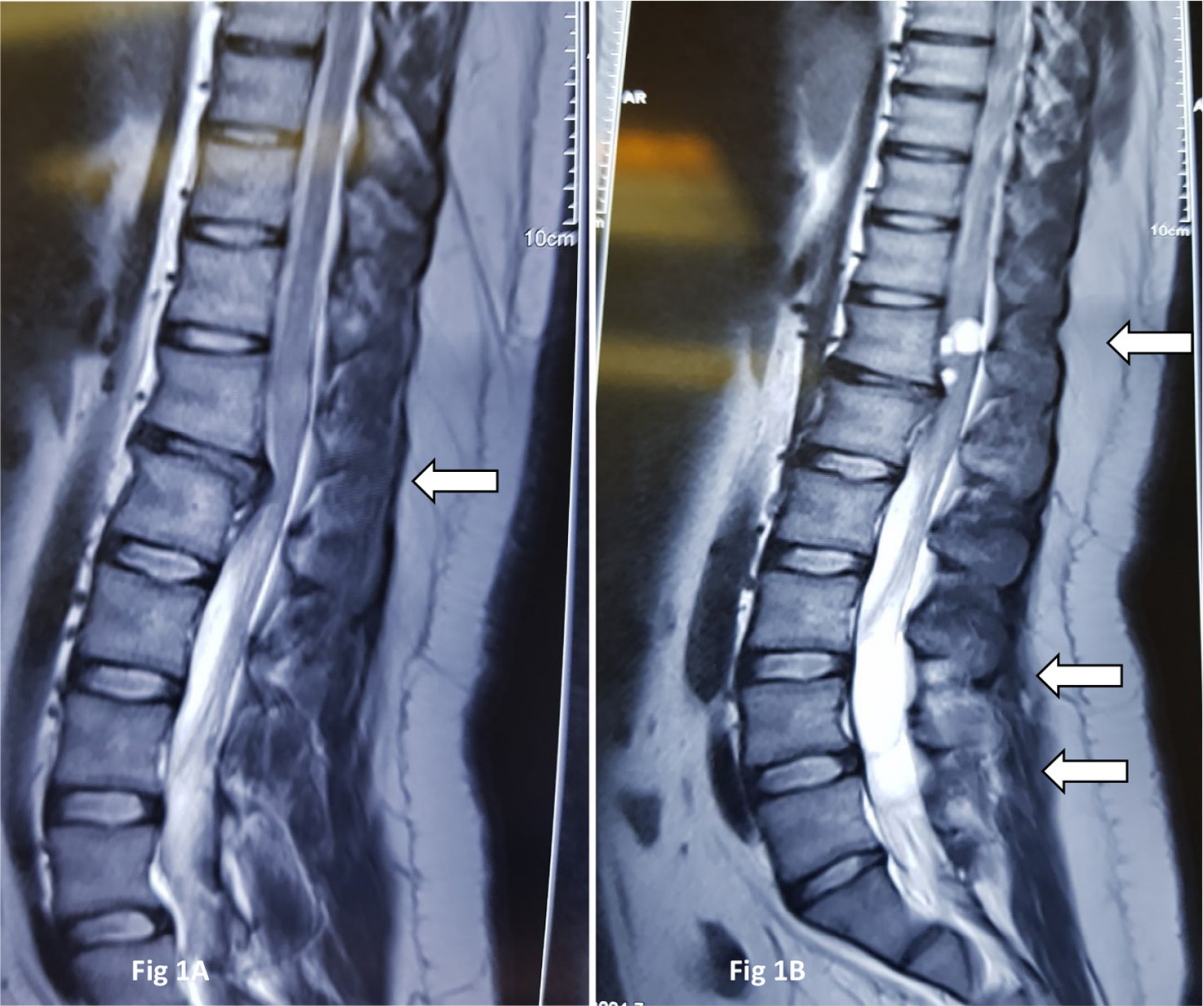
Pre‐operation imaging. (A) Sagittal T2‐weighted MRI shows extruded disc at the level of T12‐L1 (white arrow). (B) Sagittal T2‐weighted MRI shows three well‐demarcated cystic lesions with different diameters at the level of T12 vertebral body. Two other lesions with the same characteristics were present at the level of L4/L5 vertebral bodies (white arrows).

At examination, the force of the proximal lower limbs was 4 out of 5 and the distal force was 5 out of 5. Deep tendon, bulbocavernosus, and superficial anal reflexes were all intact. There was no sphincter dysfunction or hyperesthesia in the perineal area. He had hypoesthesia at L1/L2 dermatomes bilaterally. All other examinations The biochemical and hematological profiles were within normal limits and ESR and CRP levels were not elevated. Abdominopelvic ultrasonography and computed tomography (CT) scans of the chest and abdomen revealed no significant findings. In the next step, lumbosacral magnetic resonance imaging (MRI) was performed. As was predictable from the patient's symptoms, there was an extruded disc at the T12/L1 level (Figure 1A). Surprisingly, we found three well‐demarcated cystic lesions with different diameters at the level of the T12 vertebral body. Two other lesions with the same characteristics were present at the level of the L4/L5 vertebral bodies (Figure 1B). The lesions exhibited iso‐signal intensity on T1‐weighed and hyper‐signal intensity on T2‐weighed images. The cystic lesions were all intradural and possibly extramedullary at both levels. There were no signs of cord compression or bone destruction. MRI with intravenous contrast showed no enhancement.

**FIGURE 2:**
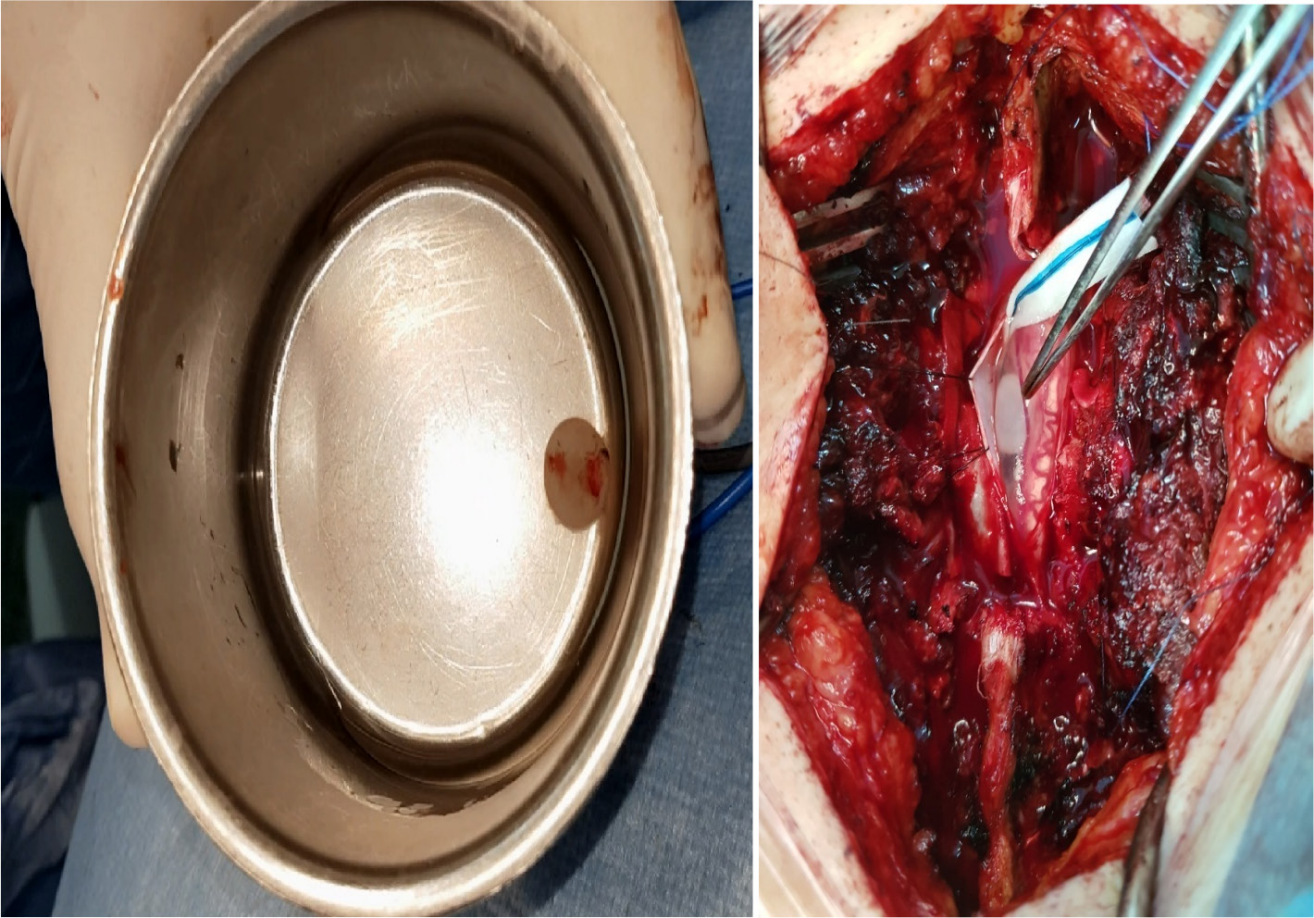
The pearl‐like intradural extramedullary cystic lesions were carefully dissected and removed.

With the pre‐operation diagnosis of an extruded T12/L1 disc and multiple spinal hydatid cysts, the patient underwent surgery in two stages. During surgery, the L4/L5 laminectomy was performed, the dura was opened and two white pearl‐like cystic masses were gently removed in order to avoid cyst rupture (Figure 2). The whole surgery field and surrounding regions were irrigated with 3% hypertonic saline. The wound was then stitched in layers.

At the next stage of the procedure, 1 week later, a T12/L1 discectomy and pedicular screw fixation (PSF) for T12 and L1 vertebrae were performed. So, the dura was opened and three white intramedullary cysts were excised without rupture. The site was then washed with hypertonic saline. At that point, the wound was closed under continuous hypertonic saline irrigation.

As a result of the operation, the patient's neurological symptoms improved, and he was discharged with anthelmintic treatment of albendazole 400 mg BD for the next 6 months.

**FIGURE 3:**
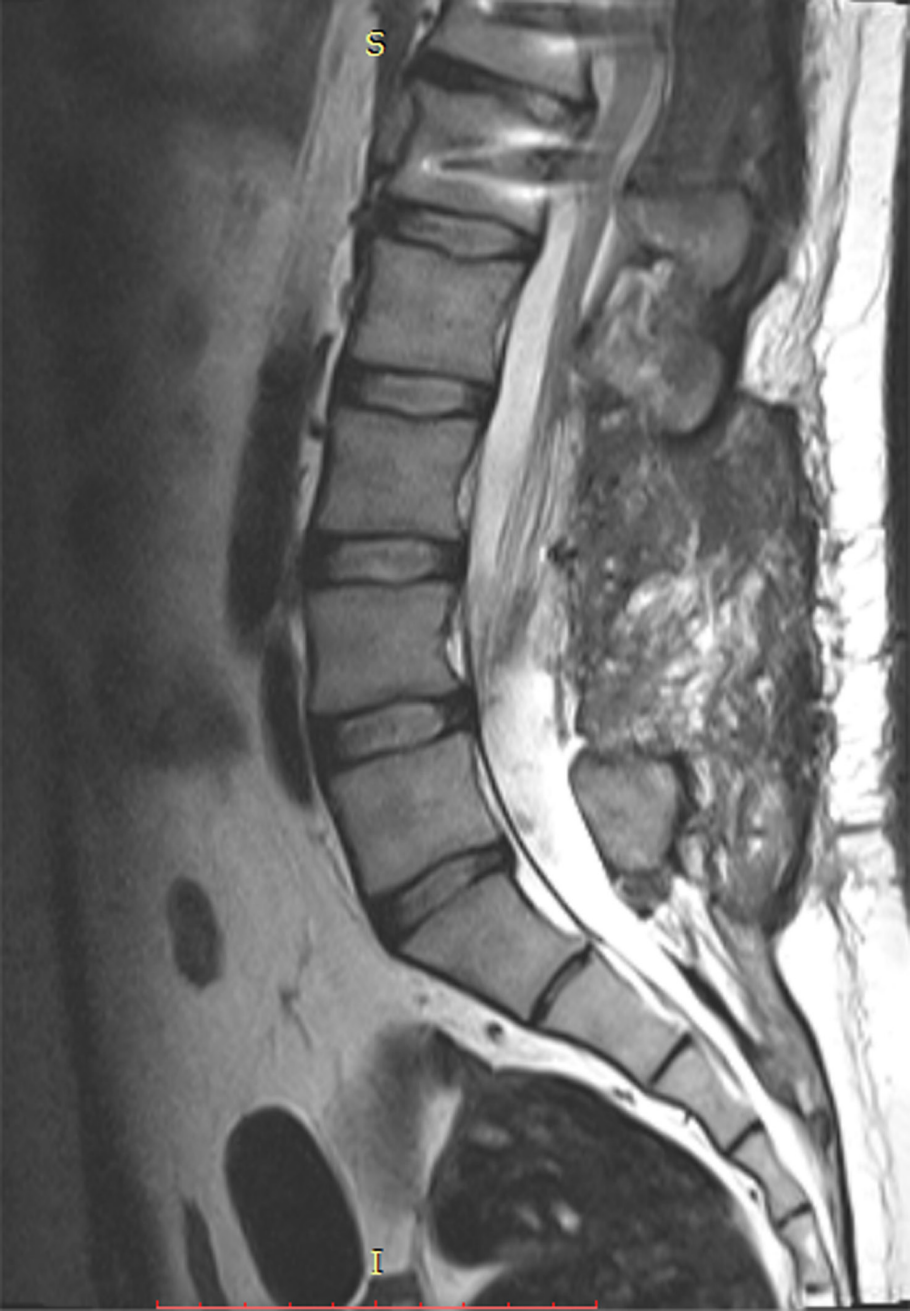
Sagittal T2‐weighted MRI 4 months after surgery shows no evidence of remnant or recurrence.

At the follow‐up session 4 months later, the patient was symptom‐free and the neuraxis MRI showed no recurrence or remnant of the CNS hydatidosis (Figure 3).

## 3 DISCUSSION

Cystic echinococcosis (CE), which affects people of all ages and genders, is a major cause of disease burden worldwide. The projected postoperative mortality rate is 2.2%, and the negatively estimated recurrence rate is 6.5%.^4^, ^5^

While it is thought to be extremely endemic in the eastern half of the Mediterranean region, central Asia, southern and Eastern Europe, northern Africa, southern America, Siberia, and western China, cystic echinococcosis is primarily observed in sheep‐raising regions. As it was completely controlled in New Zealand, Iceland, the Falkland Islands, Tasmania, and Cyprus, this illness is not known to exist in Antarctica.^4^, ^6^

Echinococcus granulosus accidentally uses humans as intermediate hosts. As permanent hosts, such as dogs and wolves, adult worms develop in their intestines and shed their eggs in their feces. The eggs are consumed by medium‐sized herbivores like sheep and cattle. Humans can get the sickness by eating food that has been contaminated with parasite eggs or by coming into close touch with carnivorous animals and their feces. The oncosphere (real larva) hatches from the egg after being consumed by humans or any other intermediate hosts, burrows into the intestinal submucosa, and then travels by veins or lymphatic vessels to internal organs such as the liver.^7^

The human liver acts as an effective barrier for most of the larvae. However, some may pass through the liver and enter the right side of the heart and then the lungs. If the larva is not lodged in the liver or lungs, it may virtually embed anywhere in the body, such as the spleen, peritoneum, heart, kidney, brain, spine, skeletal bones, and muscles.^8^ Ninety percent of the larvae are eliminated by the host reaction. However, if they survive, the metacestode or hydatid cyst develops in the affected organ over a course of years.^9^

The liver (75%), lung (15%), brain (2%–4%), and genitourinary tract (2%–3%) are often affected organs.^10^ Only 0.2%–1% of individuals have bone involvement, and the spine accounts for 50% of cases.^11^ Echinococcus primarily affects the thoracic (52%), lumbar (37%), and cervical and sacral spines.^1^, ^12^

Braithwaite and Lees divided spinal hydatid lesions into five categories: (a) main intramedullary hydatid cyst, (b) intradural extramedullary hydatid cyst, (c) extradural intraspinal hydatid cyst, (d) vertebral hydatid disease, and (e) paravertebral hydatid disease.^13^ The first three groups are quite uncommon. Comparing to extradural lesions, intradural hydatidosis mostly presents as a single cyst and at a younger age.^5^, ^14^ In our young patient, there were multiple intradural and extramedullary spinal cysts at two different levels. In addition, he had a history of prior cardiac involvement.

In a systematic analysis of 467 cases of spinal hydatidosis, Neumayr et al. reported that 78 cases (16.7%) had a history of surgical intervention for extraspinal hydatid cysts. They proposed that it is difficult to say whether spinal involvement in patients with a history of extraspinal echinococcosis results from simultaneous primary infection, or secondary hematogenous seeding or even a new exogenous contamination.^5^

Hydatid cysts are mostly misdiagnosed or missed in the early stages, as they insidiously grow for years before making any symptoms.^8^, ^11^ The condition manifests itself as significant cord compression and/or bone damage.^1^, ^7^ Symptoms and signs such as radiculopathy, myelopathy, paresthesia, paraparesis, paraplegia, sphincter malfunction, and deformity are all reported but none of them is pathognomonic of spinal hydatosis.^1^, ^5^, ^11^ Spinal hydatidosis is associated with a high degree of morbidity and mortality,^12^ and its prognosis is being compared to that of malignancies (‘*le cancer blanc’*).^5^ Interestingly, our patient presented with symptoms related to one extruded disc, but his spinal hydatid cysts were asymptomatic.

Preoperative diagnosis might be difficult since a number of illnesses resemble spinal echinococcosis. Pott's disease, spinal TB, brucellosis, osteomyelitis, mycosis, arachnoid cysts, fibrous dysplasia, simple solitary or aneurysmal bone cysts, spinal abscess, cancer, and vertebral metastases are some of the differential diagnosis listed here.^15^, ^16^

Serological assays for extra‐hepatic echinococcosis have a low diagnostic sensitivity.^3^ CT scans and x‐rays are not specific. The intervertebral disc may be intact in some cases, but the vertebral bodies may still have cystic lesions and uneven destruction.^1^, ^9^, ^11^ Finding further lesions in the liver, lungs, and other organs can be done with the aid of CT scans and ultrasonography. Other organs in our patient were examined, but no involvement was found.

The best diagnostic method for locating the cystic lesions, identifying the affected spinal levels, and establishing their relationships with neighboring organs in cases of spinal hydatidosis is magnetic resonance imaging (MRI).^11^, ^15^, ^17^ MRI images display lesions in the form of sausages with thin walls and dome‐shaped ends devoid of septations or lumen debris. Sometimes a lesion will be spherical. Hydatid cyst contents have characteristics of a CSF‐like signal. The parent cyst is iso‐intense or somewhat less intense than its fillings on T1‐weighted imaging. The T2‐weighted images show uniformly strong contents around by a dim rim.^18^ According to the histological findings, the low‐intense rim on MRI images is caused by reactive fibrosis and degeneration around the parasite membrane. Spinal hydatidosis is characterized by the presence of a significantly hypo‐intense cyst wall on T1 and T2‐weighted MRI image sequences. The survivability of the cysts can also be shown on the T2‐weighted imaging, where a cyst that has succumbed will show decreased high signal and increased low signal from collapsing cyst walls.^18^ MRI is beneficial for both early postoperative recurrence detection and assessing the efficacy of medicinal therapy 1. The diagnosis may still be supported by the histopathological results of the removed cyst.^1^

For spinal hydatidosis, the primary treatment consists of surgical excision followed by anthelmintic therapy to achieve neural decompression and establish the diagnosis.^11^ As patients usually present at advanced stages, treatment is difficult, and recurrence is common in most forms. The location of the cysts, the extent of bone involvement, and the presence of spinal instability determine the type of operative procedure, the extent of resection, and the decision whether to perform spinal stabilization or not^3^ (Pamirl. 2002). Considerable care should be taken during the surgery to avert rupture of the cysts and spillage of their content, which can cause anaphylactic reactions and/or subsequent recurrence.^11^ If cysts rupture during excision, the surgical field should be irrigated with hypertonic saline; but unfortunately recurrence is inevitable.^1^

The preferred anthelmintic drug against spinal hydatidosis is albendazole, but its efficacy and appropriate duration of treatment are still controversial.^19^ The world health organization (WHO) recommends albendazole for visceral hydatid disease, with the dosage of 10–15 mg/kg/day.^4^ Recurrence is related to the location of the cysts and is quite uncommon in the intradural extramedullary form of spinal hydatidosis, where there is no intraoperative cyst rupture.^20^, ^21^

MRI is the modality of choice in the follow‐up of spinal hydatid disease as it enables early detection of recurrences.^15^

## 4 CONCLUSION

Although spinal hydatid disease is a rare condition, its burden is remarkably high. Vertebral hydatidosis should always be considered as a differential diagnosis for typical and atypical spinal presentations, particularly in endemic areas for echinococcosis.

Preoperative neurologic evaluation combined with MRI helps in localizing the lesion and planning a suitable surgical approach. The treatment consists of surgery and adjuvant anthelmintic therapy. The cysts should be excised carefully to avoid rupture and subsequent recurrence. Irrigation of the surgical field with hypertonic saline is also helpful.

Strict follow‐up and regular MRIs are necessary to detect recurrence at early stages. Despite medical advances, many aspects of spinal hydatidosis are still vaguely understood. Therefore, further studies in this field are warranted.

## AUTHOR CONTRIBUTIONS

**Seyyed Mostafa Ahmadi:** Conceptualization; investigation; supervision; validation; visualization; writing – original draft. **Keyvan Tayebi Meibodi:** Conceptualization; investigation; methodology; supervision; writing – original draft. **Neda Raeesi:** Conceptualization; data curation; methodology; supervision; validation. **Mohammad Ali Bitaraf:** Investigation; supervision; writing – review and editing. **Arad Iranmehr:** Conceptualization; supervision; visualization; writing – original draft; writing – review and editing.

## FUNDING INFORMATION

This research did not receive any specific grant from funding agencies in the public, commercial, or not‐for‐profit sectors.

## CONFLICT OF INTEREST STATEMENT

The authors report no conflict of interest to declare.

## CONSENT

Written informed consent was obtained from the patient to publish this report in accordance with the journal's patient consent policy.

## Data Availability

All of the data related to this manuscript are available by request from the corresponding author.
